# Association between bicuspid aortic valve phenotype and patterns of valvular dysfunction: A meta‐analysis

**DOI:** 10.1002/clc.23736

**Published:** 2021-11-03

**Authors:** Zhenzhen Mai, Lina Guan, Yuming Mu

**Affiliations:** ^1^ Department of Echocardiography First Affiliated Hospital of Xinjiang Medical University Urumqi China

**Keywords:** aortic regurgitation, aortic stenosis, bicuspid aortic valve, meta‐analysis

## Abstract

**Background:**

Valvular dysfunction is a common complication in patients with bicuspid aortic valves (BAV). The aim of this study was to determine the relationship between BAV morphology patterns and valve dysfunction.

**Methods:**

We searched the PubMed, The Cochrane Library, Web of Science, and CNKI until May 31, 2020, to identify all studies investigating the morphology of BAV and valvular dysfunction, and data were extracted according to the Preferred Reporting Items for Systematic reviews and Meta‐Analyses (PRISMA). Data were analyzed using Stata 15.1 software. The additional characteristics (gender, mean age) were collected to perform a meta‐regression analysis.

**Results:**

Thirteen studies on BAV‐RL (*n* = 2002) versus BAV‐RN (*n* = 1254) and raphe (*n* = 4001) versus without raphe (*n* = 673) were included. The BAV‐RL patients showed a higher incidence of aortic regurgitation than BAV‐RN patients (OR = 1.46; 95% CI: 1.12 to 1.90, *p* = .005), while the BAV‐RL patients showed a lower incidence of aortic stenosis than BAV‐RN patients (OR = 0.66, 95% CI: 0.58 to 0.76, *p* = .000); BAV patients with raphe presents a higher incidence of aortic regurgitation than those without raphe (OR = 1.95, 95% CI: 1.12–3.39, *p* = .017). No differences were found between raphe and without raphe group in the incidence of aortic stenosis (OR = 0.97, 95% CI: 0.53 to 1.76, *p* = .907). Mean age and gender had no influence on observed differences.

**Conclusions:**

Our results confirmed a relationship between different BAV phenotypes and aortic valve dysfunction. BAV‐RL and BAV with raphe are more likely to develop aortic regurgitation, while patients with BAV‐RN present a higher possibility to develop aortic stenosis.

## 1 INTRODUCTION

The bicuspid aortic valve (BAV) is the most common congenital cardiac defect that observed in 1%–2% of general population,^1^ with a male to female ratio of about 3:1. Patients with BAV are at a high risk of developing aortic valve dysfunction, either stenosis or regurgitation, or both. Studies have suggested that 33% of patients with BAV will suffer serious and life‐threatening complications in their lifetime. Therefore, early detection and prevention of the complications caused by BAV are of paramount importance.^2^ BAV appears to be inherited in an autosomal dominant fashion with incomplete penetrance. It has been postulated that the defective genes encoding the protein matrix structure could be responsible for developmental impairment of heart, and leading to valvular abnormalities.^3^, ^4^, ^5^ BAV presents several phenotypes, and an animal experiment demonstrated that different BAV phenotypes are caused by different developmental processes, suggesting that different BAV phenotypes should be considered as different etiological entities with different valvular lesions, aortic size, and elasticity.^6^ Thus, more credit should be given to the association between BAV phenotypes with valvular dysfunction.^7^

The most common BAV pattern is fusion of the right and left coronary cusps, and fusion of the right and noncoronary cusps.^8^, ^9^ Previous evidence suggests that various BAV types, distinguished by the morphology of the valve cusp fusion, may carry different relationships with valvular dysfunction; however, the published literature is incoherent in this regard. Several studies have reported an increased frequency of significant valvulopathy in pediatric patients with right and left coronary cusps fusion,^10^ while another longitudinal follow‐up study claimed that BAV phenotype failed to demonstrate a prognostic implication.^11^

## 2 AIM OF THE STUDY

Therefore, the purpose of our study was to evaluate the impact of different BAV cusp fusion morphology on the incidence of valvular dysfunction, and provide clues and evidence for early clinical diagnosis and prevention of complications.

## 3 METHODS

### 3.1 Search strategy

**FIGURE 1:**
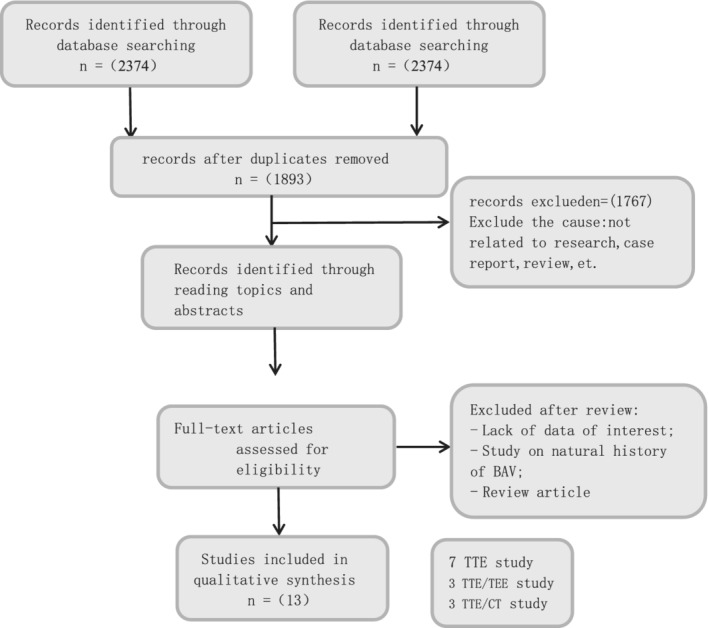
Flow diagram for study selection. This flow chart shows the initial search results and final review of 11studies after consideration of exclusions

A systematic search was performed in the electronic databases (PubMed, The Cochrane Library, Web of Science, and CNKI), using the following search terms in all possible combinations: bicuspid aortic valve, aortic regurgitation, aortic stenosis, valve dysfunction. Articles were rejected on initial screening if from the title or the abstract it was judged that the article does not report aortic valve dysfunction and BAV morphology. Subsequently, the full text of the remaining articles was retrieved. All the references were also scanned. The particular studies were examined to exclude duplicated and overlapped data. Finally, only studies evaluating aortic stenosis and aortic regurgitation were included. In case of missing data, the authors were contacted by e‐mail to try to retrieve the original data. Each article was analyzed by two independent individuals and data extraction was done independently. In case of disagreement, a third investigator was consulted. Discrepancies were resolved by consensus. Data extraction was conducted according to the Preferred Reporting Items for Systematic reviews and Meta‐Analyses (PRISMA) (Figure 1).^12^

### 3.2 Study selection and data extraction

Inclusion criteria were as follows: (1) the domestic and foreign published literature, all studies that participants were diagnosed as BAV by TTE or TEE or CT; (2) An information about the morphology of BAV (RL morphology and RN morphology) according to previously mentioned definition and information related to valve dysfunction. (3) More complete raw data is available in the literature for calculation of odds ratio (OR).

Exclusion criteria were as follows: (1) No data on the BAV morphology and valve dysfunction, or there is not enough data available for odds ratio (OR) calculations; (2) Review article, case studies, animal experiments, and conference abstracts. (3) Research that not able to access the full text through various channels is only an abstract.

The following data were also extracted from each study: first author, year of publication, used imaging modality, study population characteristics including mean age, male gender percentage, sample size (number of subjects in particular BAV subtypes), number of patients with AS, and number of patients with AR.

### 3.3 Statistical analyses and risk of bias assessment

The presented meta‐analysis was performed using Statistica 15.1. The frequency variable is expressed as *n* (%). Differences among AS and AR between the two types of BAV patients were expressed as odds ratio (OR) with pertinent 95% CI for dichotomous variables. Overall effect was tested using Z scores, and significance was set at *p* < .05. Statistical heterogeneity among studies was assessed with the chi‐square Cochran's Q test and with the I^2^ statistic, which measures the inconsistency across study results and describes the proportion of total variation in study estimates. To evaluate the individual impact of each study on the overall effect size, sensitivity analysis was conducted using the leave‐one‐out approach, by estimating the weighted mean difference in the absence of each single study.

The presence of publication bias was evaluated using Egger's weighted regression tests and Begg's rang correlation. Publication bias was evaluated visually by inspection of funnel plots of *SE* and mean difference asymmetry. Visual inspection of funnel plot asymmetry was performed to address for possible small‐study effect, as well as the Egger test to address publication bias, over and above any subjective evaluation. *p* < .05 was considered statistically significant.^13^

## 4 RESULTS

### 4.1 Search results and study characteristics

The total of 2376 articles were searched from PubMed, The Cochrane Library, Web of Science, and CNKI. Articles that abstracts and titles were irrelevant to our objection were excluded during the initial screening. Then, full texts of 23 articles were analyzed. At last, 13^14^, ^15^, ^16^, ^17^, ^18^, ^19^, ^20^, ^21^, ^22^, ^23^, ^24^, ^25^, ^26^ articles meet the inclusion criteria. The number of patients varied from 67 to 785, mean age ranged from 17.2 to 59 years, and the prevalence of male sex is from 60% to 78%. The following imaging modalities were used in analyzed studies: transthoracic echocardiography (TEE), transesophageal echocardiography (TTE), computed tomography (CT), and complex MDCT/TEE imaging. The baseline characteristics of the all included studies are presented in Tables 1 and 2.

**TABLE 1:** Description of the studies included into the analysis of AS associated with BAV morphology

Reference	Time	Imaging	Country	RL		RN		Mean age (y)	Man (%)
				AS	N	AS	N		
Sun^21^	2017	TTE/TEE	Korea	269	361	292	320	59 ± 12	62.0
Ruzmetov^17^	2015	TTE	US	27	96	56	114	17.2 ± 9.9	60.0
Ren XS^24^	2017	TTE	China	37	125	49	74	50.3 ± 3.8	65.5
Miśkowiec^15^	2016	TTE/TEE	Poland	26	46	15	21	55.3 ± 6.7	78.0
Tabriziet^19^	2018	TTE/TEE	Iran	51	188	63	112	40 ± 16	72.0
Kang^14^	2013	TTE/CT	Korea	43	93	49	74	54.6 ± 4.4	68.9
Hong^22^	2014	TTE/CT	Korea	33	192	37	80	51.7 ± 4.4	72.7
Huang^16^	2013	TTE	Singapore	27	117	25	74	48.4 ± 5.8	67.0
Tuluce^20^	2017	TTE/TEE	Turkey	42	105	33	49	37 (17–70)	71.4
Wei Liqun^23^	2018	TTE	China	55	89	103	141	52.6 ± 5.0	51.6
Evangelist^18^	2017	TTE	Spain	146	590	58	195	47.4 ± 6.8	70.2
				**AR**	**N**	**AR**	**N**		
Sun^21^	2017	TTE/TEE	Korea	144	361	71	320	59 ± 12	62.0
Ruzmetov^17^	2015	TTE	US	40	96	60	114	17.2 ± 9.9	60.0
Ren XS^24^	2017	TTE	China	74	125	13	74	50.3 ± 3.8	65.5
Miśkowiec^15^	2016	TTE/TEE	Poland	42	46	18	21	55.3 ± 6.7	78.0
Tabriziet^19^	2018	TTE/TEE	Iran	150	188	77	112	40 ± 16	72.0
Kang^14^	2013	TTE/CT	Korea	31	93	5	74	54.6 ± 4.4	68.9
Hong^22^	2014	TTE/CT	Korea	58	192	11	80	51.7 ± 4.4	72.7
Huang^16^	2013	TTE	Singapore	49	117	26	74	48.4 ± 5.8	67.0
Tuluce^20^	2017	TTE/TEE	Turkey	75	105	36	49	37 (17–70)	71.4
Wei Liqun^23^	2018	TTE	China	44	89	39	141	52.6 ± 5.0	51.6
Evangelist^18^	2017	TTE	Spain	146	590	44	195	47.4 ± 6.8	70.2

**TABLE 2:** Description of the studies included into the analysis of valvular dysfunction associated with BAV morphology (Raphe+ vs. Raphe−)

Reference	Time	Imaging	Country	Raphe+		Raphe−		Mean age (y)	Man (%)
				AS	N	AS	N		
Kong^25^	2017	TTE	Netherlands	721	1881	51	237	47.0 ± 8.0	72.0
Sievers^26^	2014	NA	Germany	550	1247	64	115	54.2 ± 3.4	76.7
Ren XS^24^	2017	TTE	China	25	109	61	88	50.3 ± 3.8	65.5
Hong^22^	2014	TTE/CT	Korea	50	120	50	89	51.7 ± 4.4	72.7
Evangelist^18^	2017	TTE	Spain	166	644	16	144	47.4 ± 6.8	72.0
				**AR**	**N**	**AR**	**N**		
Kong^25^	2017	TTE	Netherlands	144	361	71	320	47.0 ± 8.0	72.0
Sievers^26^	2014	NA	Germany	40	96	60	114	54.2 ± 3.4	76.7
Ren XS^24^	2017	TTE	China	74	125	13	74	50.3 ± 3.8	65.5
Hong^22^	2014	TTE/CT	Korea	42	46	18	21	51.7 ± 4.4	72.7
Evangelist^18^	2017	TTE	Spain	150	188	77	112	47.4 ± 6.8	72.0

Abbreviations: AR, aortic regurgitation (at least moderate); AS, aortic stenosis (at least moderate); CT, computed tomography; NA, data not available; RL, right and left cusp fusion bicuspid aortic valve morphology; RN, right and noncoronary cusp fusion bicuspid aortic valve morphology; TTE, transthoracic echocardiography.

### 4.2 Correlation between BAV morphology(RL and RN)and aortic stenosis

**FIGURE 2:**
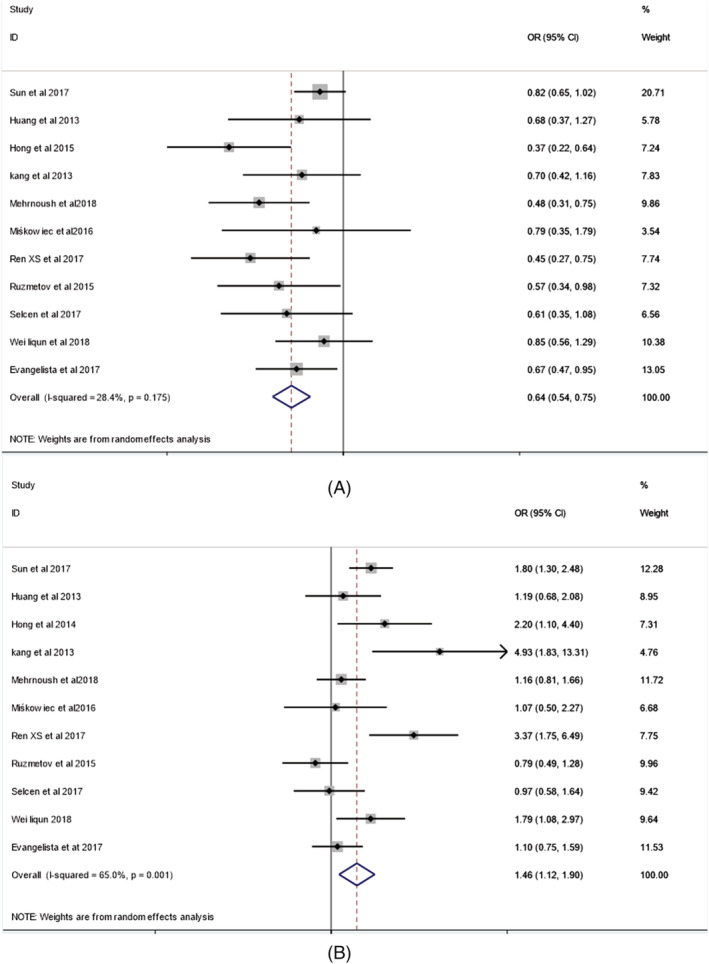
Forest plots. (A) Incidence of Aortic stenosis in patients with RL BAV and RN BAV; (B) Incidence of aortic regurgitation in patients with RL BAV and RN BAV. RL = right and left cusp fusion of bicuspid aortic valve; RL = right or left and noncoronary cusp fusion of bicuspid aortic valve; 95% CI = 95% confidence interval

Eleven studies,^14^, ^15^, ^16^, ^17^, ^18^, ^19^, ^20^, ^21^, ^22^, ^23^, ^24^ including 2002 BAV‐RL and 1254 BAV‐RN patients, showed that AS was reported in 37.8% BAV‐RL subjects and in 62.1% BAV‐RN patients (OR = 0.66; 95% CI: 0.58 to 0.76; *p* = .000), the difference was statistically significant. Heterogeneity among studies was not significant (I^2^ = 28.4%; *p* = .124). The combined effect quantity OR was determined using a fixed effect model. Fixed effects meta‐regression suggests that age (slope: 0.01; 95% CI: −0.01–0.03; Z = −0.59; *p* = .396), male gender (slope: −0.02; 95% CI: −0.04–0.01; Z = 0.46; *p* = .122) does not associated with the incidence of aortic regurgitation. Forest plot summarizing the meta‐analysis of studies comparing aortic stenosis between RL and RN BAV groups is shown in Figure 2A.

### 4.3 Correlation between BAV morphology (RL and RN) and aortic regurgitation

In 11 studies,^14^, ^15^, ^16^, ^17^, ^18^, ^19^, ^20^, ^21^, ^22^, ^23^, ^24^ including 2002 BAV‐RL and 1254 BAV‐RN patients, AR was reported in 42.6% of BAV‐RL subjects and 31.9% BAV‐RN patients (OR = 1.46; 95% CI: 1.12 to 1.90; *p* = .005), the difference was statistically significant. Heterogeneity among studies was significant (I^2^ = 65.0%; *p* = 0.001). Random‐effects meta‐regression suggests that age (slope: 0.02; 95% CI:−0.01–0.05; Z = −0.59; *p* = .215), male gender (slope: 0.01; 95% CI: −0.05–0.06; Z = −0.59; *p* = .767) does not associated with the incidence of aortic regurgitation. Forest plot summarizing the meta‐analysis of studies comparing aortic regurgitation between RL and RN BAV groups is shown in Figure 2B.

### 4.4 Correlation between BAV morphology (raphe vs. without raphe) with aortic regurgitation

**FIGURE 3:**
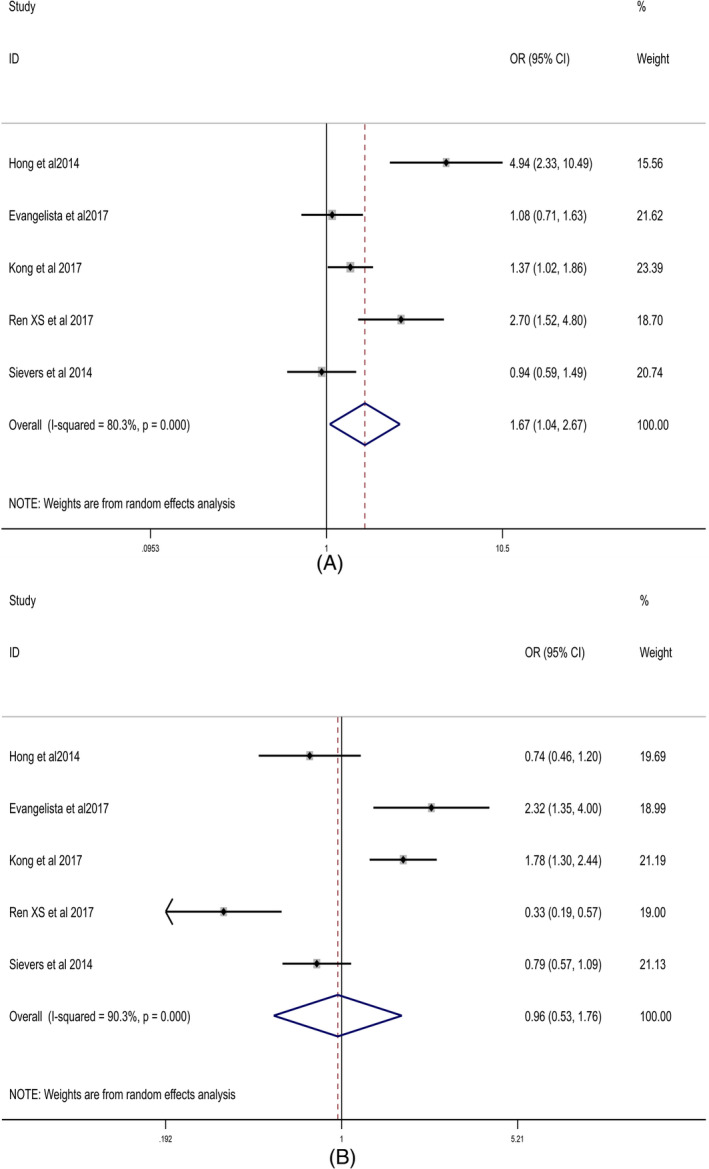
Forest plots. (A) Forest plot diagram of correlation between bicuspid aortic valve classification (Raphe vs. Nonraphe) and aortic regurgitation (AR); (B). Forest plot diagram of correlation between bicuspid aortic valve classification (Raphe vs. Nonraphe) and aortic stenosis (AS)

Five studies,^18^, ^22^, ^24^, ^25^, ^26^ including 4674 patients, exhibit 4001 (85.6%) BAV patients has raphe, and 673 (14.4%) BAV patients are without raphe. Bicuspid aortic valves with raphe had a higher frequency to develop aortic regurgitation (28.9% vs. 21.4%; OR = 1.67; 95% CI: 1.04–2.67, *p* = .032). Heterogeneity among studies was significant (I^2^ = 80.3%, *p* = .001), the combined effect quantity OR was determined using Random effect model. Forest plot summarizing the meta‐analysis of studies comparing aortic regurgitation between raphe and without raphe BAV groups is shown in Figure 3A.

### 4.5 Correlation between BAV morphology (raphe vs. without raphe) with aortic stenosis

Five studies^18^, ^22^, ^24^, ^25^, ^26^ demonstrated that bicuspid aortic valves with or without raphe do not affect the incidence of developing aortic stenosis (37.8% vs. 36.0%, OR = 0.96, 95% CI: 0.53–1.76, *p* = .907). Heterogeneity among studies was significant (I^2^ = 90.3%; *p* = 0.000), the combined effect quantity OR was determined using Random effect model. Forest plot summarizing the meta‐analysis of studies comparing aortic stenosis between raphe and without raphe BAV groups is shown in Figure 3B.

### 4.6 Sensitivity analysis

Presented pooled results were found to be robust in the performed leave‐one‐out sensitivity analysis, removing 1 study at a time. Obtained stability of the presented results confirms a significant difference in the frequency of aortic stenosis and aortic regurgitation between the BAV‐RL and BAV‐RN groups. For the analysis of the association between BAV phenotype and aortic stenosis, I^2^ ranged from 8.5% to 42.7%, showing increased heterogeneity (Table S1). For the analysis of the association between BAV phenotype and aortic regurgitation, I^2^ ranged from 60.0% to 69.5%, the results did not differ from the previous ones. (Table S2).

### 4.7 Publication bias analysis

Because it is recognized that publication bias can affect results of meta‐analyses, we attempted to assess this potential bias using funnel plot visual analysis. Our results suggest that there is no potential bias for the comparison of BAV‐RL and BAV‐RN in aortic stenosis and aortic regurgitation. The Begg rank correlation test (Kendall tau with continuity correction: Pr > |z| = 0.53, Z = 0.62) and the Egger linear regression test (intercept: −1.71, 95% CI: −3.7 to 0.35; t = −1.88, *p* > |t| = .093) exhibit no evidence of publications bias when comparing the incidence of aortic stenosis between BAV‐RL and BAV‐RN patients. Moreover, the Begg rank correlation analysis (Kendall tau with continuity correction: Pr > |z| = 0.35, Z = 0.93), and the Egger linear regression test (intercept: 1.8, 95% CI: −1.87 to 5.62; t = 1.13, *p* > |t| = 0.287) suggested also no evidence of publications bias when comparing the incidence of aortic stenosis between BAV‐RL and BAV‐RN patients.

## 5 DISCUSSION

Our meta‐analysis shows that BAV patients with right and left cusp fusion are incline to develop aortic regurgitation, while patients with right and noncoronary cusp fusion are more likely to develop aortic stenosis. Moreover, bicuspid aortic valves with raphe showed a higher incidence of aortic regurgitation. However, with or without raphe does not affect the incidence of aortic stenosis. This meta‐analysis is the first to assess the effect of BAV phenotype on valvular dysfunction differences.

BAV has diverse morphologic variants, and might result in different pathogenesis and clinical manifestations, the BAV phenotype has been an interesting topic for many investigators. There are multiple classifications of the pathological types of bicuspid aortic valve malformations, and the most common classification is based on the presence or absence of fused spine formation, leaflet fusion type, and leaflet spatial location. This study classifies the presence or absence of fused spine formation and type of leaflet fusion.

The most common complication of the BAV in adults is AV dysfunction necessitating surgical aortic repair or AV replacement (AVR) (population‐based 25‐year risk of AVR is up to 53%),^27^ and is most commonly driven by presence of severe AS followed by severe aortic regurgitation (AR) and mixed AV disease. As a cause of AVR, AS has been reported between 61% and 88% in population‐based studies and studies from tertiary‐referral centers, conversely, AR is only responsible for 15 and 29% AV surgery.^28^ Some studies have suggested a BAV phenotype role in the rapid progression of valvular dysfunction.^10^ Because of the early and rapid progression of valvular lesions in patients with BAV malformation, the age of the patients undergoing surgery is about 10 years younger than the normal population. In addition, analysis of BAV morphology is of prognostic relevance, Fernandes et al.^10^ demonstrated a 64% free from intervention in patients with fusion of the R‐N. However, in patients with fusion of the R‐L, 91% free from intervention was noted. Therefore, the purpose of our meta‐analysis was to evaluate the impact of BAV morphology on valve dysfunction, use morphological differences to predict the trend of complications.

Because a raphe is commonly seen in patients with BAV, the clinical significance of raphe is of interest. A global registry showed that the presence of a visible raphe is associated with significant (moderate or greater) AS and AR and higher future incidence of AVR.^25^ Furthermore, BAV patients with raphe had higher rates of AVR compared with patients without raphe.^25^ It is reported that the raphe of a BAV and a higher tendency for calcium deposition are important causes of significant valve dysfunction.^29^ Therefore, patients with BAV with raphe tend to develop significant valvular dysfunction at a younger age. Our meta‐analysis found that aortic regurgitation was more frequently observed among patients with raphe.

In addition, evaluation of inter‐ethnic differences in valve morphology and function in patients with BAV is important for the worldwide spread of transcatheter aortic valve replacement (TAVR). Kong et al.^30^ reported that there is significant heterogeneity in BAV across European and Asian population, type 0 (without raphe) is more frequently observed in Europeans and fusion raphe between the right and the noncoronary cusps is more frequently observed in Asians. The European group had higher incidence of significant aortic regurgitation than the Asian group (44.2% vs. 26.8%, respectively; *p* < .001). There was no difference in the grades of aortic stenosis between these two populations.

BAV patients show obvious heterogeneity in many different clinical aspects, including the BAV phenotype and the severity of valve dysfunction. From a clinical point of view, our study confirms some practical implications. When studying BAV patients, the imaging should be not only focused on the type of valvular dysfunction, but should be also performed a very careful scrutiny of the BAV phenotype, seeking all the spectrum of aortic valve malformations, having in mind that BAV phenotype can directly affect the type of valvular dysfunction. Information about BAV morphology may help to facilitate more individualized patient management and risk stratification. Further study is necessary to determine why the distribution of valvular dysfunction differed according to BAV morphology.

The present meta‐analysis has some limitations. First, due to the limited number of studies included, the heterogeneity of the studies about the relationship between the BAV and aortic regurgitation is greater, and the source of heterogeneity is not further analyzed. However, we have at least partially reduced the effect of observed heterogeneity on the overall effect size by selecting a random effects model analysis. Second, there is inevitably a risk shift in the study of any population, especially the confounder in retrospective studies. These differences may be responsible for the heterogeneity between studies. Third, a systematic approach to the detailed classification of BAV should be routinely applied in clinical practice to provide new insights into this common disease entity in the future.

Our results confirmed a relationship between different BAV phenotypes and aortic valve dysfunction. BAV‐RL and BAV with raphe are more likely to develop aortic regurgitation, while patients with BAV‐RN present a higher possibility to develop aortic stenosis.

## CONFLICT OF INTEREST

The authors declare no potential conflict of interests.

## Supporting information


**Appendix** S1: Supporting Information

## Data Availability

The data used to support the findings of this study are available from the corresponding author upon request.
