# Supplementary material for: Association between bicuspid aortic valve phenotype and patterns of valvular dysfunction: A meta‐analysis
Source: Clin Cardiol. 2021 Nov 3;44(12):1683–91. doi: 10.1002/clc.23736 (PMC8715397; doi:10.1002/clc.23736)
Supplement: Supplementary file 1 — Appendix S1: Supporting Information [file CLC-44-1683-s001.doc]

**Supplemental Materials**

**Supplemental Tables**

**Supplemental Table 1** Leave-one-out sensitivity analysis of the BAV morphology impact on the aortic stenosis

| **Reference** | **Quantitative data synthesis**     **Heterogeneity analysis**  **RL(n)**   **RN(n)**  **Effect size**  **95% CI**    **Z value P value Q**   **df(Q)**  **I^2^ (%)** | | | | | | | | |
| --- | --- | --- | --- | --- | --- | --- | --- | --- | --- |
| Overall effect | 2002 | 1254 | 0.66 | 0.58-0.76 | 6.21 | 0.17 | 13.96 | 10 | 28.4 |
| Leave-one-out sensitivity analysis | | | | | | | | | |
| Sun et al 2017 | 1641 | 934 | 0.60 | 0.51-0.70 | 6.36 | 0.42 | 9.15 | 9 | 1.6 |
| Huang et al 2013 | 1975 | 1180 | 0.67 | 0.58-0.76 | 6.09 | 0.12 | 13.9 | 9 | 35.5 |
| Hong et al 2015 | 1810 | 1174 | 0.69 | 0.60-0.79 | 5.52 | 0.42 | 9.17 | 9 | 1.9 |
| kang et al 2013 | 1909 | 1180 | 0.66 | 0.58-0.76 | 6.06 | 0.12 | 13.9 | 9 | 35.4 |
| Mehrnoush et al2018 | 1814 | 1138 | 0.69 | 0.60-0.78 | 5.49 | 0.23 | 11.7 | 9 | 23.0 |
| Miśkowiec et al2016 | 1956 | 1233 | 0.66 | 0.58-0.76 | 6.20 | 0.13 | 13.8 | 9 | 34.7 |
| Ren XS et al 2017 | 1887 | 1180 | 0.68 | 0.60-0.78 | 5.62 | 0.24 | 11.5 | 9 | 21.9 |
| Ruzmetov et al 2015 | 1906 | 1140 | 0.67 | 0.59-0.77 | 5.88 | 0.14 | 13.6 | 9 | 34.0 |
| Selcen et al 2017 | 1897 | 1205 | 0.67 | 0.59-0.76 | 5.99 | 0.13 | 13.9 | 9 | 35.2 |
| Wei Liqun et al 2018 | 1913 | 1113 | 0.65 | 0.57-0.74 | 6.28 | 0.18 | 12.58 | 9 | 28.5 |
| Evangelista et al 2017 | 1412 | 1059 | 0.67 | 0.58-0.76 | 5.79 | 0.13 | 13.9 | 9 | 35.5 |

**Supplemental Table 2** Leave-one-out sensitivity analysis of the BAV morphology impact on the aortic regurgitation

| **Reference** | **Quantitative data synthesis**     **Heterogeneity analysis**  **RL(n)**   **RN(n)** **Effect size** **95% CI**   **Z value P value Q**   **df(Q)**  **I^2^ (%)** | | | | | | | | |
| --- | --- | --- | --- | --- | --- | --- | --- | --- | --- |
| Overall effect | 2002 | 1254 | 1.46 | 1.12-1.90 | 2.83 | 0.001 | 28.6 | 10 | 65.0 |
| Leave-one-out sensitivity analysis | | | | | | | | | |
| Sun et al 2017 | 1641 | 934 | 1.42 | 1.07-1.90 | 2.39 | 0.002 | 25.5 | 9 | 64.7 |
| Huang et al 2013 | 1975 | 1180 | 1.49 | 1.12-1.99 | 2.76 | 0.001 | 28.3 | 9 | 68.2 |
| Hong et al 2015 | 1810 | 1174 | 1.41 | 1.08-1.86 | 2.48 | 0.001 | 26.9 | 9 | 66.5 |
| kang et al 2013 | 1909 | 1180 | 1.37 | 1.07-1.71 | 2.52 | 0.008 | 22.2 | 9 | 59.5 |
| Mehrnoush et al2018 | 1814 | 1138 | 1.46 | 1.12-1.90 | 2.83 | 0.001 | 28.6 | 10 | 65.0 |
| Miśkowiec et al2016 | 1956 | 1233 | 1.49 | 1.13-1.98 | 2.84 | 0.001 | 28.1 | 9 | 68.0 |
| Ren XS et al 2017 | 1887 | 1180 | 1.35 | 1.06-1.72 | 2.40 | 0.012 | 21.2 | 9 | 57.6 |
| Ruzmetov et al 2015 | 1906 | 1140 | 1.55 | 1.19-2.02 | 3.30 | 0.006 | 22.9 | 9 | 60.7 |
| Selcen et al 2017 | 1897 | 1205 | 1.52 | 1.15-2.02 | 2.96 | 0.002 | 26.7 | 9 | 66.2 |
| Wei Liqun et al 2018 | 1913 | 1113 | 1.43 | 1.08-1.91 | 2.47 | 0.001 | 27.6 | 9 | 67.4 |
| Evangelista et al 2017 | 1412 | 1059 | 1.52 | 1.14-2.05 | 2.82 | 0.002 | 26.8 | 9 | 66.4 |
